# An Efficient User Authentication and User Anonymity Scheme with Provably Security for IoT-Based Medical Care System

**DOI:** 10.3390/s17071482

**Published:** 2017-06-23

**Authors:** Chun-Ta Li, Tsu-Yang Wu, Chin-Ling Chen, Cheng-Chi Lee, Chien-Ming Chen

**Affiliations:** 1 Department of Information Management, Tainan University of Technology, 529 Zhongzheng Road, Tainan 71002, Taiwan; th0040@mail.tut.edu.tw; 2 Fujian Provincial Key Laboratory of Big Data Mining and Applications, Fujian University of Technology, Fuzhou 350118, China; 3 National Demonstration Center for Experimental Electronic Information and Electrical Technology Education, Fujian University of Technology, 3 Xueyuan Road, Fuzhou 350118, China; 4 Department of Computer Science and Information Engineering, Chaoyang University of Technology, 168 Jifeng East Road, Taichung 41349, Taiwan; 5 School of Information Engineering, Changchun University of Technology, Changchun 130600, China; 6 Department of Library and Information Science, Fu Jen Catholic University, 510 Jhongjheng Road, New Taipei 24205, Taiwan; cclee@mail.fju.edu.tw; 7 Department of Photonics and Communication Engineering, Asia University, 500 Lioufeng Road, Taichung 41354, Taiwan; 8 Harbin Institute of Technology Shenzhen Graduate School, Shenzhen University Town, Xili, Nanshan District, Shenzhen 518055, China; chienming.taiwan@gmail.com

**Keywords:** elliptic curve cryptography (ECC), Internet of Things (IoTs), medical care system, smart cards, user authentication, wireless sensor networks (WSNs)

## Abstract

In recent years, with the increase in degenerative diseases and the aging population in advanced countries, demands for medical care of older or solitary people have increased continually in hospitals and healthcare institutions. Applying wireless sensor networks for the IoT-based telemedicine system enables doctors, caregivers or families to monitor patients’ physiological conditions at anytime and anyplace according to the acquired information. However, transmitting physiological data through the Internet concerns the personal privacy of patients. Therefore, before users can access medical care services in IoT-based medical care system, they must be authenticated. Typically, user authentication and data encryption are most critical for securing network communications over a public channel between two or more participants. In 2016, Liu and Chung proposed a bilinear pairing-based password authentication scheme for wireless healthcare sensor networks. They claimed their authentication scheme cannot only secure sensor data transmission, but also resist various well-known security attacks. In this paper, we demonstrate that Liu–Chung’s scheme has some security weaknesses, and we further present an improved secure authentication and data encryption scheme for the IoT-based medical care system, which can provide user anonymity and prevent the security threats of replay and password/sensed data disclosure attacks. Moreover, we modify the authentication process to reduce redundancy in protocol design, and the proposed scheme is more efficient in performance compared with previous related schemes. Finally, the proposed scheme is provably secure in the random oracle model under ECDHP.

## 1. Introduction

As more network technologies and smart devices have been developed, many IoT (Internet of Things) applications have been proposed, such as transportation and logistics services, healthcare services and a variety of smart environment (home, office, plant) domains. IoT is going to create a world where physical things can be seamlessly integrated into communication networks in order to provide autonomous and intelligent services for improving human beings’ life. In general, the IoT system involves three components: a sensing unit contains a large number of sensors, actuators and mobile terminals to sense physical environments; a network layer includes all network techniques with heterogeneous network configurations for data transmission; intelligent computing offers expected services or applications to IoT end users by mining and analyzing data processors.

IoT-based wireless sensor networks have been getting considerable attention from a variety of domains, such as environmental monitoring, intelligent appliances in daily living, medical care services, etc. Due to the ranking of the most common diseases in advanced countries having changed to chronic and cardiovascular diseases, the demands for medical care of such patients have increased substantially in hospitals and healthcare institutions. For the development of medical care services in hospitals and healthcare institutions, IoT-based WSNs technology is used to supplement physiological collection and measurement, enabling doctors, caregivers and families to examine the physiological conditions of patients remotely at anytime and anyplace through the Internet [1,2,3,4,5,6]. On the basis of IoT employed for medical care service in hospitals or healthcare institutions, WSNs enable sensing and collecting the physiological parameters of patients periodically, transmitting the acquired data to the authorized medical personnel, enabling professional doctors and medical personnel to monitor patients’ health conditions in real time and providing patients with appropriate medical care and medical treatment.

To apply IoT-based WSNs to medical care services successfully, ensuring the personal privacy of patients and preventing malicious network intrusion are paramount. Undoubtedly, the foundation of security is to authenticate the legitimacy of remote users and ensure the integrity of data transmissions [7,8,9,10,11,12]. In the last decade, a diversity of user authentication schemes in WSNs have been presented. In 2006, Wong et al. [13] introduced an efficient user authentication scheme for WSNs using lightweight hashing functions and XOR operations. In 2007, Tseng et al. [14] pointed out the vulnerability of Wong et al.’s scheme to replay, forgery and password guessing attacks. Furthermore, in 2008, Lee [15] showed that the computational overheads of Wong et al.’s scheme are not suitable for resource-constrained sensor nodes. In 2009, Das [16] suggested a two-factor (namely the password and smart card) authentication mechanism for WSNs, which not only prevents a series of security threats, but also achieves efficiency in terms of computational overheads. However, Huang et al. [17] and Li et al. [18] pointed out the vulnerability of Das’s scheme to off-line password guessing, user impersonation, node impersonation and unknown user attacks and that it does not provide the property of user anonymity. In 2012, Yoo et al. [19] pointed out the vulnerability of Huang et al.’s scheme to insider and parallel session attacks and that it does not provide mutual authentication between system participants. In 2013, Xue et al. [20] presented a temporal-credential-based authentication scheme for resource-constrained WSNs, and the authors claimed that their scheme provides relatively more security criteria without increasing system overheads too much in terms of communication, computation and storage. Parallel to Xue et al.’s work, in the same year, Li et al. [3] cryptanalyzed that Xue et al.’s scheme cannot withstand off-line password guessing, stolen-verifier, privileged insider, many logged-in users’ and stolen smart card attacks, and the above security threats make Xue et al.’s scheme inapplicable to practical WSN applications.

**Figure 1:**
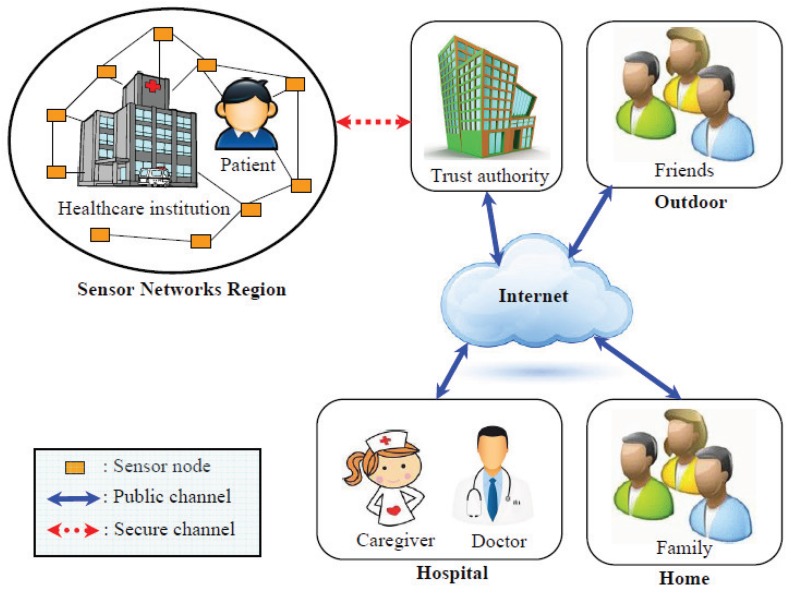
The IoT-based medical care system [21].

In order to design a secure and two-factor user authentication scheme for wireless healthcare sensor networks, Liu and Chung [21] in 2016 proposed a bilinear pairing-based [22] authentication scheme, and Figure 1 illustrates the comprehensive structure of the IoT-based medical care system, which could be applied in hospitals or healthcare institutions. When patients live in hospitals or healthcare institutions, they wear smart clothes in which body sensors are embedded in the piece of clothing and collect their physiological parameters (such as blood pressure, heartbeat, body pulse, electrocardiography and body temperature). Therefore, the users (such as doctors, caregivers, families and friends) in the medical care system can remotely inquire and monitor physiological information on patients with the help of trust authority. Before accessing the system, users must register with the trusted authority in person. After successful registration, the trusted authority issues a smart card to the user, and he/she can then use his/her smart card and mobile devices (such as smart phone, PDA, laptop and tablet computer) to log into the medical care system. After successful authentication, the user can access the sensed data of patients measured from sensor nodes within a limited time. Nevertheless, in this paper, we present a cryptanalysis of Liu–Chung’s authentication scheme and indicate that their scheme is susceptible to the password disclosure, replay, sensed data disclosure, sensed data forgery, off-line password guessing and stolen smart card attacks. To solve the above-mentioned security problems, we present an improved version of Liu–Chung’s authentication scheme using ECC, and we prove that the proposed scheme is secure under the elliptic curve discrete logarithm problem (ECDLP) and the elliptic curve Diffie–Hellman problem (ECDHP). In addition, by designing the mechanism of dynamic identity in the authentication process, we can build an extended scheme with user anonymity. User anonymity [23,24,25] means that a remote user’s real identity will be masked during the login session, and he/she cannot be linked or traced by any outsiders. Furthermore, the correctness of mutual authentication between participants has been proven in the random oracle model under ECDHP. Finally, the proposed scheme requires lower computational overheads compared with other ECC-based schemes, and this advantage makes our scheme more suitable and practical for IoT-based medical care systems.

The rest of the paper is organized as follows. In Section 2, a brief review of Liu–Chung’s authentication scheme is provided. In Section 3, security weaknesses developed to attack Liu–Chung’s scheme are presented. In Section 4, the improved scheme is proposed. Security and performance analyses of our proposed scheme are presented in Section 5 and Section 6, respectively. Section 7 concludes this paper.

## 2. Review of Liu–Chung’s Authentication Scheme

This section briefly reviews Liu–Chung’s authentication scheme [21], and their scheme consists of five phases, including: setup phase, registration phase, login phase, verification phase and access control and encryption phase. For convenience of description, the terminology and notations used in the paper are summarized as follows:

- $U_{i}$: The user.
- TA: The trusted authority.
- *S*: The sensor nodes deployed in hospitals and healthcare institutions.
- $ID_{i}$: The identity of $U_{i}$.
- $PW_{i}$: The password of $U_{i}$.
- h(·): A one-way hash function.
- $\hat{e}\left(a,b\right)$: The bilinear pairing function using parameter *a* and parameter *b*.
- *a*: A private parameter generated by TA.
- $T_{L}$: The login time of $U_{i}$.
- $T_{now}$: The current time.
- $T_{u}$: The time limit on the legal access to *S* by the user $U_{i}$.
- ΔT: The transmission delay.
- *m*: The sensed data collected from *S*.
- ||: The message concatenation.
- ⊕: The XOR operation.

### 2.1. Setup Phase

In this phase, the trusted authority TA selects a bilinear map $\hat{e}:G_{1}\times G_{1}\to G_{2}$ and $P_{0}\in G_{1}$ and generates two one-way hash functions $H_{1}:{\left\{0,1\right\}}^{*}\to G_{2}$ and $H_{2}:G_{2}\to {\left\{0,1\right\}}^{*}$, where $G_{1}$ is an additive cyclic group of points on an elliptic curve *E* over $F_{p}$, $G_{2}$ is a multiplicative cyclic group of a finite field $F_{p}^{*}$ and *p* is a large prime, such that q|p-1 for some great prime *q*. Then, TA selects the secret key $S_{0}\in Z_{q}^{*}$ and publishes the parameter $P_{pub}=S_{0}\times P_{0}$.

### 2.2. Registration Phase

In this phase, the user registers with the trusted authority TA through a secure channel to be a legal user. The details of registration phase are as follows:

Step 1: $U_{i}$ registers an authenticated identity $ID_{i}$ with TA and sets password $PW_{i}$.
Step 2: $U_{i}$ sends $<ID_{i},PW_{i}>$ to TA.
Step 3: TA computes $Q_{priv}=S_{0}\times U_{pub}$, where $U_{pub}=U_{priv}\times P_{0}$ and $U_{priv}\in Z_{q}^{*}$ are $U_{i}$’s public parameter and secret key, respectively.
Step 4: TA stores the parameters $<h\left(\cdot \right),Q_{priv},ID_{i},PW_{i},a>$ in $U_{i}$’s smart card, where *a* represents a private parameter generated by TA and all of the sensor nodes of TA include *a*.
Step 5: TA issued the smart card to $U_{i}$.

### 2.3. Login Phase

In this phase, the user inserts his/her smart card into the device and inputs $ID_{i}$ and $PW_{i}$. Then, the smart card performs the following steps:

Step 1: The smart card checks the $ID_{i}$ and $PW_{i}$ entered by $U_{i}$ matches those stored in the smart card. If yes, the smart card executes Step 2. Otherwise, the smart card terminates this phase.
Step 2: The smart card computes $r=h(ID_{i}||PW_{i}||a)$ and $Sig=r\times Q_{priv}$.
Step 3: The smart card sends $<Sig,r,T_{L},ID_{i}>$ to TA through a public channel, where $T_{L}$ represents $U_{i}$’s login time to the TA.

### 2.4. Verification Phase

When TA receives the login request $<Sig,r,T_{L},ID_{i}>$ from $U_{i}$, TA authenticates $U_{i}$ through the following steps:

Step 1: TA checks the validity of $ID_{i}$ and verifies if $\hat{e}\left(P_{0},Sig\right)=\hat{e}\left(P_{pub},r\times U_{pub}\right)$. If yes, TA approves the request of $U_{i}$ and executes Step 2. If no, TA rejects the request of $U_{i}$.
Step 2: TA checks if $T_{now}-T_{L}<\Delta T$. If yes, TA executes Step 3. Otherwise, it means that the login time exceeds the transmission delay, and the login request is rejected by TA.
Step 3: TA generates a random number *b* and computes $E=h(b⊕U_{pub})$. Then, TA sends *E* to $U_{i}$ through a public channel.
Step 4: TA sends $<T_{u},b,ID_{i}>$ to all of the sensor nodes *S* through a secure channel and notifies *S* that $U_{i}$ is legal. Note that $T_{u}$ represents the time limit on the legal access to sensor node data by $U_{i}$.

### 2.5. Access Control and Encryption Phase

When the user $U_{i}$ is authenticated as legal, $U_{i}$ can legally access sensed data *m* in *S* within a limited time, and $U_{i}$ and *S* perform the following steps:

Step 1: $U_{i}$ inserts his/her smart card into the device and inputs $ID_{i}$ and $PW_{i}$. Then, the smart card verifies whether $ID_{i}$ and $PW_{i}$ inputted matches the data stored in the card. If yes, the smart card executes Step 2.
Step 2: The smart card computes $C=h(a||ID_{i})⊕E$.
Step 3: The smart card sends $<C,ID_{i},T^{\prime }>$ to *S* through a public channel, where $T^{\prime }$ represents a timestamp.
Step 4: Upon receiving $<C,ID_{i},T^{\prime }>$ from $U_{i}$, *S* verifies if $T_{now}-T^{\prime }<\Delta T$ and $T_{now}=T_{u}$. If yes, *S* executes Step 4.
Step 5: *S* computes $C^{\prime }=h(a||ID_{i})⊕h\left(b⊕U_{pub}\right)$ and checks whether $C=C^{\prime }$. If yes, the sensed data *m* will be transmitted, and *S* executes Step 5. If no, *S* terminates this session.
Step 6: *S* computes $M=m⊕H_{2}\left(\hat{e}\left(U_{pub},P_{pub}\right)\right)$.
Step 7: *S* sends *M* to $U_{i}$ through a public channel.
Step 8: $U_{i}$ uses the secret parameter $Q_{priv}$ and the public parameter $P_{0}$ to perform the following calculation to obtain *m*:
  $$\begin{array}{ccc} m & = & M⊕H_{2}\left(\hat{e}\left(Q_{priv},P_{0}\right)\right)\\ & = & m⊕H_{2}\left(\hat{e}\left(U_{pub},P_{pub}\right)\right)⊕H_{2}\left(\hat{e}\left(Q_{priv},P_{0}\right)\right)\\ & = & m⊕H_{2}\left(\hat{e}\left(U_{pub},P_{pub}\right)\right)⊕H_{2}\left(\hat{e}\left(S_{0}\times U_{pub},P_{0}\right)\right)\\ & = & m⊕H_{2}\left(\hat{e}\left(U_{pub},P_{pub}\right)\right)⊕H_{2}\left(\hat{e}{\left(U_{pub},P_{0}\right)}^{S_{0}}\right)\\ & = & m⊕H_{2}\left(\hat{e}\left(U_{pub},P_{pub}\right)\right)⊕H_{2}\left(\hat{e}\left(U_{pub},S_{0}\times P_{0}\right)\right)\\ & = & m⊕H_{2}\left(\hat{e}\left(U_{pub},P_{pub}\right)\right)⊕H_{2}\left(\hat{e}\left(U_{pub},P_{pub}\right)\right)\\ & = & m \end{array}$$

**Figure 2:**
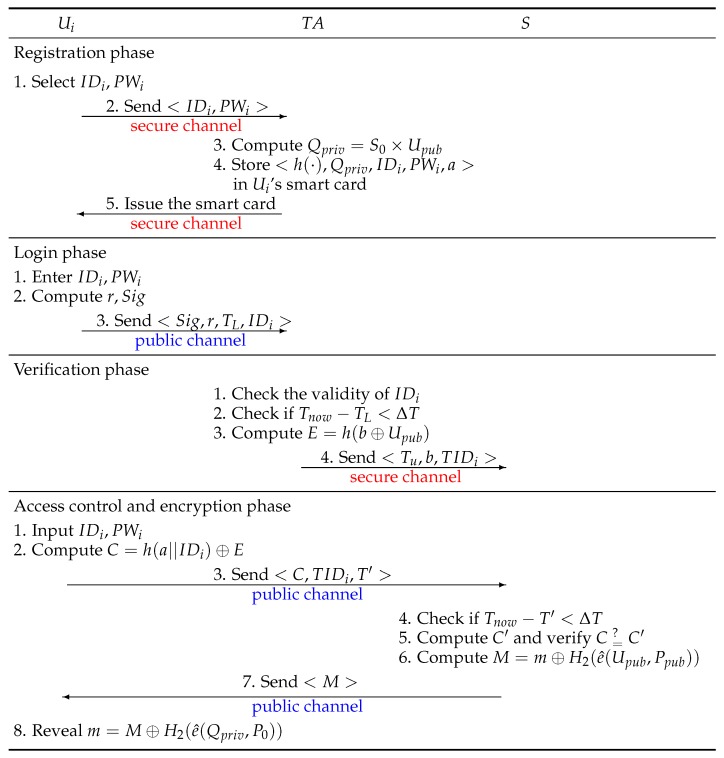
The schematic of Liu–Chung’s authentication scheme for IoT-based medical care system.

Figure 2 shows the schematic of Liu–Chung’s authentication scheme for the IoT-based medical care system.

## 3. Weaknesses of Liu–Chung’s Authentication Scheme

In this section, we present the security weaknesses of Liu–Chung’s scheme. We show that their scheme has some security problems and that an attacker $U_{a}$ can mount different types of attacks on Liu–Chung’s scheme.

### 3.1. Password Disclosure Attacks

In real environments, the user may register with a number of remote services by using a common password PW and the identity ID for his/her convenience. Thus, the privileged-insider of TA may try to use the knowledge of user’s PW and ID to access another remote services. In the registration phase of Liu–Chung’s scheme, $U_{i}$ registers to TA by sending $\left(ID_{i},PW_{i}\right)$. Therefore, $U_{i}$’s sensitive password $PW_{i}$ will be revealed by the privileged-insider of TA.

### 3.2. Replay Attacks

In the login phase of Liu–Chung’s scheme, although the transmitted login message $<Sig,r,T_{L},ID_{i}>$ includes timestamp $T_{L}$, however, the other login parameters $<Sig,r,ID_{i}>$ of $U_{i}$ are unchanged. Thus, an attacker $U_{a}$ could replay the eavesdropped messages, such as $U_{i}$’s login request $<Sig,r,T_{L}^{\prime },ID_{i}>$ with $U_{a}$’s current login time $T_{L}^{\prime }$. Finally, $U_{a}$ can bypass the timestamp checking and replay attacks cannot prevented in Liu–Chung’s scheme.

### 3.3. Sense Data Disclosure Attacks

In the access control and encryption phase of Liu–Chung’s scheme, the sensor node *S* sends the encrypted sensed data *M* to $U_{i}$ through an insecure channel. Due to the public $U_{pub}$ of $U_{i}$ and the public $P_{pub}$ of TA, once an attacker $U_{a}$ eavesdrops the encrypted sensed data *M* from the public channel, $U_{a}$ can perform the following calculation to obtain *m* without knowing $Q_{priv}$:

$$\begin{array}{ccc} m & = & M⊕H_{2}\left(\hat{e}\left(U_{pub},P_{pub}\right)\right)\\ & = & m⊕H_{2}\left(\hat{e}\left(U_{pub},P_{pub}\right)\right)⊕H_{2}\left(\hat{e}\left(U_{pub},P_{pub}\right)\right)\\ & = & m \end{array}$$

Finally, Liu–Chung’s scheme cannot prevent sensed data disclosure attacks.

### 3.4. Sense Data Forgery Attacks

In the access control and encryption phase, we found that Liu–Chung’s scheme allows the attacker $U_{a}$ to forge a fake sensed data $m^{\prime }$ for the user $U_{i}$, and $U_{i}$ wrongly believes he/she has received the physiological conditions of the patients. The sensed data forgery attacks on Liu–Chung’s scheme are as follows:

(1) When the sensor node *S* sends $M=m⊕H_{2}\left(\hat{e}\left(U_{pub},P_{pub}\right)\right)$ to the user $U_{i}$, $U_{a}$ intercepts the message *M*.
(2) $U_{a}$ maliciously forges a fake sensed data $m^{\prime }$ and computes $M^{\prime }=m^{\prime }⊕H_{2}\left(\hat{e}\left(U_{pub},P_{pub}\right)\right)$, where $U_{pub}$ and $P_{pub}$ are public parameters of $U_{i}$ and TA, respectively. Then, $U_{a}$ sends $M^{\prime }$ to the user $U_{i}$.
(3) Upon receiving the message $M^{\prime }$, $U_{i}$ uses the secret parameter $Q_{priv}$ and the public parameter $P_{0}$ to obtain $m^{\prime }=M^{\prime }⊕H_{2}\left(\hat{e}\left(Q_{priv},P_{0}\right)\right)$.

Therefore, the attacker $U_{a}$ can control the sensed data that occur between the user $U_{i}$ and the sensor nodes *S*.

### 3.5. Stolen Smart Card Attacks

Usually, the smart card of the user $U_{i}$ is equipped with tamper-resistant hardware. However, if $U_{i}$’s smart card is lost or stolen, the attacker $U_{a}$ may obtain all of the sensitive parameters stored in its memory by monitoring the power consumption of the smart card [26]. Assume that $U_{a}$ obtains the smart card of $U_{i}$ and extracts the parameters $<h\left(\cdot \right),Q_{priv},ID_{i},PW_{i},a>$ stored inside it. $U_{a}$ then can make a valid login request with ease. For example, $U_{a}$ uses h(·), $ID_{i}$, $PW_{i}$, *a* and $Q_{priv}$ and computes $r=h(ID_{i}||PW_{i}||a)$ and $Sig=r\times Q_{priv}$. Finally, $U_{a}$ can make a valid login request to impersonate $U_{i}$ by sending $<Sig,r,T_{L}^{\prime },ID_{i}>$ to the trusted authority TA, where $T_{L}^{\prime }$ is the current login time of $U_{a}$.

### 3.6. Off-Line Password Guessing Attacks

Since Liu–Chung’s authentication scheme is executed in the open network environment, then we assumed that an attacker $U_{a}$ can eavesdrop the communication channels between $U_{i}$ and TA in the login phase. Moreover, we assumed that $U_{a}$ was a legitimate user in the medical care system, and he/she can extract the parameter *a* by launching power analysis attack [26]. Thus, $U_{a}$ could guess $U_{i}$’s password through the following steps.

(1) $U_{a}$ eavesdrops the message $<Sig,r,T_{L},ID_{i}>$ sent by a legal user $U_{i}$, where $r=h(ID_{i}||PW_{i}||a)$.
(2) $U_{a}$ guesses a password $PW_{a}$ and computes $r_{a}=h(ID_{i}||PW_{a}\left||a\right)$ in an off-line manner.
(3) $U_{a}$ checks whether $r_{a}$ is equal to *r* or not. If it is equal, $U_{i}$’s sensitive password is successfully guessed. Otherwise, $U_{a}$ repeats Steps (1) and (2) until the correct password is found.

From the above descriptions, we conclude that $U_{a}$ could derive $U_{i}$’s password through an off-line manner, and Liu–Chung’s authentication scheme could not succeed against the off-line password guessing attacks.

## 4. The Proposed Scheme

**Figure 3:**
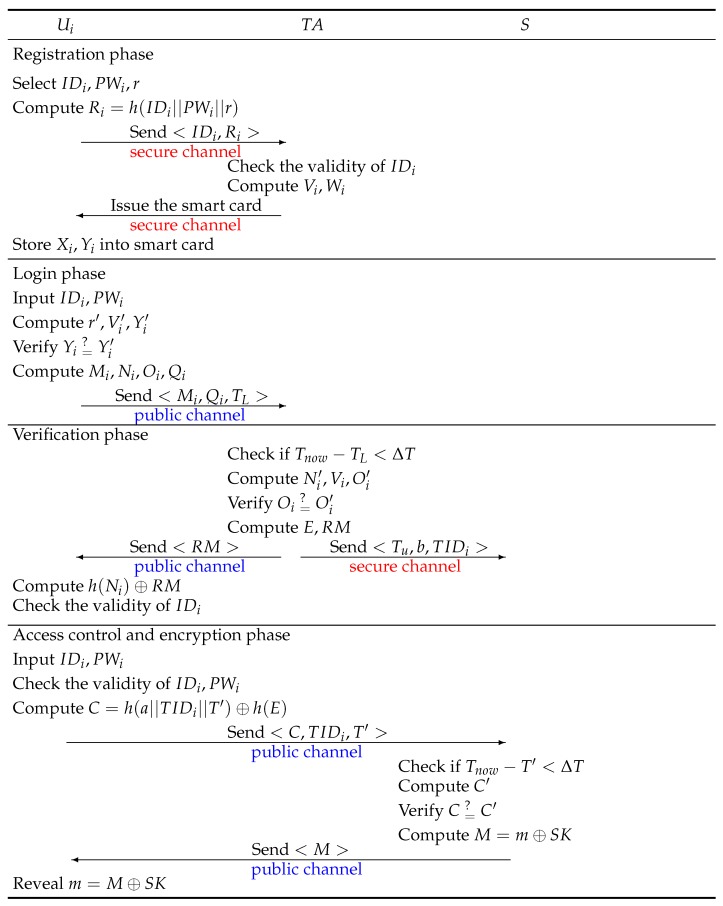
The schematic of our proposed scheme for IoT-based medical care system.

This section proposes the new and improved lightweight user authentication scheme for medical care tailored for the Internet of Things environment. The proposed scheme is based on Liu–Chung’s scheme; thus, it tackles and eliminates all of the previously-mentioned security problems and vulnerabilities of their scheme. As Liu–Chung’s scheme, the proposed scheme also consists of five phases: setup, registration, login, verification and access control and encryption. Figure 3 shows the schematic of our proposed scheme for the IoT-based medical care system. 

### 4.1. Setup Phase

In this phase, the trusted authority TA selects an elliptic curve *E* over $F_{p}$ and a base point $P_{0}$ over the *E* and chooses a secure one-way hashing function $h\left(\cdot \right):{\left\{0,1\right\}}^{*}\to {\left\{0,1\right\}}^{l}$, where *p* is a large prime such that q|p-1 for some great prime *q* and *l* means the length of the output. In addition, TA chooses the secret key $S_{0}\in Z_{q}^{*}$ and computes its public key $P_{pub}=S_{0}\times P_{0}$. Finally, TA keeps $S_{0}$ securely and publishes $<E,q,P_{0},P_{pub},h\left(\cdot \right)>$ as system parameters.

### 4.2. Registration Phase

In this phase, the user registers with the trusted authority TA through a secure channel to be a legal user, and the details of registration phase are as follows:

Step 1: $U_{i}$ registers an authenticated identity $ID_{i}$ and password $PW_{i}$ with TA and chooses a random number *r* for computing $R_{i}=h(ID_{i}||PW_{i}\left||r\right)$.
Step 2: $U_{i}$ sends the registration request $<ID_{i},R_{i}>$ to TS through a secure channel.
Step 3: TA checks whether $ID_{i}$ has been registered or not. If $ID_{i}$ has not been registered, TA computes $V_{i}=h(ID_{i}\left|\right|S_{0}\left||a\right)$ and $W_{i}=V_{i}⊕R_{i}$. Then, TA stores the parameters $<W_{i},a,E,q,P_{0},P_{pub},h\left(\cdot \right)>$ in $U_{i}$’s smart card and issued the smart card to $U_{i}$, where *a* represents a private parameter generated by TA and all the sensor nodes of TA include *a*.
Step 4: $U_{i}$ computes $V_{i}=W_{i}⊕h(ID_{i}||PW_{i}\left||r\right)$, $X_{i}=r⊕h(ID_{i}||PW_{i})$ and $Y_{i}=h(V_{i}||r||h(ID_{i}||PW_{i}))$ and stores $<X_{i},Y_{i}>$ into the smart card. Finally, $U_{i}$’s smart card contains the parameters $<Y_{i},X_{i},W_{i},a,E,q,P_{0},P_{pub},h\left(\cdot \right)>$.

### 4.3. Login Phase

In this phase, the user inserts his/her smart card into the device and inputs $ID_{i}$ and $PW_{i}$. Then, the smart card executes the following steps:

Step 1: The smart card checks the $ID_{i}$ and $PW_{i}$ entered by $U_{i}$ matches those stored in the smart card. First, the smart card computes $r^{\prime }=X_{i}⊕h(ID_{i}||PW_{i})$, $V_{i}^{\prime }=W_{i}⊕h(ID_{i}||PW_{i}\left|\right|r^{\prime })$ and $Y_{i}^{\prime }=h(V_{i}^{\prime }\left|\right|r^{\prime }||h(ID_{i}||PW_{i}))$ and verifies whether $Y_{i}=Y_{i}^{\prime }$. If it holds, the smart card executes Step 2. Otherwise, the smart card terminates this phase.
Step 2: The smart card generates a random number α and computes $M_{i}=\alpha \times P_{0}$, $N_{i}=\alpha \times P_{pub}$, $O_{i}=h(ID_{i}\left|\right|V_{i}^{\prime }\left|\right|T_{L})$ and $Q_{i}=h\left(N_{i}\right)⊕(ID_{i}\left|\right|O_{i})$ and sends $<M_{i},Q_{i},T_{L}>$ to TA through a public channel, where $T_{L}$ represents $U_{i}$’s login time to the TA.

### 4.4. Verification Phase

When TA receives the login request $<M_{i},Q_{i},T_{L}>$ from $U_{i}$, TA authenticates $U_{i}$ through the following steps:

Step 1: TA checks if $T_{now}-T_{L}<\Delta T$. If yes, TA executes Step 2. Otherwise, it means that the login time exceeds the transmission delay, and the login request will be rejected by TA.
Step 2: TA computes $N_{i}^{\prime }=S_{0}\times M_{i}$ and $(ID_{i}\left|\right|O_{i})=Q_{i}⊕h\left(N_{i}^{\prime }\right)$ and checks if user’s $ID_{i}$ is recorded by TA. If yes, TA executes Step 3. Otherwise, the login request is denied by TA.
Step 3: TA goes on to compute $V_{i}=h(ID_{i}\left|\right|S_{0}\left||a\right)$ by using the identity $ID_{i}$ and checks that the decrypted $O_{i}$ is the same as computed $O_{i}^{\prime }=h(ID_{i}\left|\right|V_{i}\left|\right|T_{L})$. If no, the session is aborted by TA. Otherwise, TA computes $E=h(b⊕TID_{i})$ and $RM=h\left(N_{i}^{\prime }\right)⊕(ID_{i}||TID_{i}\left|\right|T_{u}\left||E\right)$ sends the response message <RM> to $U_{i}$ through a public channel, where *b* represents a random number and $TID_{i}$ represents a temporary identity for the user $U_{i}$.
Step 4: TA sends $<T_{u},b,TID_{i}>$ to all of the sensor nodes *S* via a secure channel and notifies *S* that the temporary identity $TID_{i}$ is legal in the next access control and encryption phase.
Step 5: When $U_{i}$ receives <RM> from TA, $U_{i}$ authenticates TA by computing $(ID_{i}||TID_{i}\left|\right|T_{u}\left||E\right)=h\left(N_{i}\right)⊕RM$ and checks that the decrypted $ID_{i}$ is involved in RM or not. If yes, $U_{i}$ confirms that TA is legal and the parameters $TID_{i}$, $T_{u}$ and *E* will be used in access control and encryption phase. Otherwise, $U_{i}$ ends this session. Note that $TID_{i}$ and *E* must be kept secret by $U_{i}$ and temporarily stored into $U_{i}$’s smart card until the end of the access control and encryption phase.

### 4.5. Access Control and Encryption Phase

When the user $U_{i}$ is authenticated as legal, $U_{i}$ can legally access sensed data *m* in *S* within a permitted time $T_{u}$, and $U_{i}$ and *S* perform the following steps:

Step 1: In this step, the executed operations are the same as Step 1 of the login phase.
Step 2: The smart card calculates $C=h(a||TID_{i}||T^{\prime })⊕h\left(E\right)$ and sends $<C,TID_{i},T^{\prime }>$ to *S* through a public channel, where $T^{\prime }$ represents a timestamp.
Step 3: Upon receiving $<C,TID_{i},T^{\prime }>$ from $U_{i}$, *S* verifies if $T_{now}-T^{\prime }<\Delta T$ and $T_{now}\subseteq T_{u}$. If yes, *S* executes Step 4.
Step 4: *S* computes $C^{\prime }=h(a||TID_{i}\left|\right|T^{\prime })⊕h\left(h\left(b⊕TID_{i}\right)\right)$ by using the *b* transmitted by TA and the temporary identity $TID_{i}$ of the user to examine whether $C=C^{\prime }$. If yes, the validity of $U_{i}$ is authenticated by *S*, and the sensed data *m* will be transmitted by *S*. If no, *S* terminates this session.
Step 5: *S* computes the session key $SK=h(E⊕a⊕T_{u})$ and encrypts the sensed data by computing M=m⊕SK. Then, *S* sends <M> to $U_{i}$ through a public channel. Note that the session key SK provides a secure channel for protecting data transmission between *S* and $U_{i}$.
Step 6: When $U_{i}$ receives <M> from *S*, $U_{i}$ uses the parameters $\left(E,a,T_{u}\right)$ to calculate the session key $SK=h(E⊕a⊕T_{u})$ and decrypts the sensed data *m* by computing m=M⊕SK.

Note that SK should be frequently updated when $U_{i}$’s $T_{u}$ is expired. If so, $U_{i}$ returns to the login and verification phases for requesting a new $T_{u}$ with TA. Finally, a new SK will be established and updated among $U_{i}$ and *S* in the access control and encryption phase.

## 5. Security Analysis of the Proposed Scheme

In this section, we analyze the security of our proposed scheme, and show that it is able to prevent the above-mentioned weaknesses in Liu–Chung’s scheme. The security of the proposed scheme is based on the collision-free one-way hash function and two hard problems: the elliptic curve discrete logarithm problem (ECDLP) and the elliptic curve Diffie–Hellman problem (ECDHP), defined as follows:

ECDLP: Given a base point *P* over an elliptic curve *E* and a random variable $b\in Z_{q}^{*}$, it is computationally infeasible to find out an integer solution *a* such that b=aP.
ECDLP: Given three parameters $P,aP,bP\in Z_{q}^{*}$, it is computationally infeasible to compute $abP\in Z_{q}^{*}$.

We analyze and summarize the main security advantages of our proposed scheme as follows.

### 5.1. Resistance to Password Disclosure and Password Guessing Attacks

In the registration phase, the user’s password $PW_{i}$ is used in the message $R_{i}=h(ID_{i}||PW_{i}\left||r\right)$. Although the privileged-insider of TA can obtain the message $R_{i}$ and the identity $ID_{i}$ of the user, it is unable to know the user’s sensitive password $PW_{i}$ due to *r* being randomly selected by the user, and $PW_{i}$ is protected by $h(ID_{i}||PW_{i}||r)$. Note that deriving $PW_{i}$ from $h(ID_{i}||PW_{i}||r)$ is equal to implementing the brute-force attack to crack the one-way hashing function. Moreover, during the login, verification and access control and encryption phases, neither the smart card nor the transmitted messages include user’s password $PW_{i}$. Hence, the proposed scheme eliminates the possibility of password disclosure and password guessing attacks.

### 5.2. Resistance to Replay Attacks

The timestamps and random numbers are common countermeasures to prevent replay attacks in the authentication process. Since the messages $<M_{i},Q_{i},T_{L}>$ and $<C,TID_{i},T^{\prime }>$ contain freshly generated timestamps $T_{L}$ and $T^{\prime }$ and these timestamps are also embedded in the protected messages $Q_{i}=h\left(N_{i}\right)⊕(ID_{i}||h(ID_{i}\left|\right|V_{i}^{\prime }\left|\right|T_{L}))$ and $C=h(a||TID_{i}||T^{\prime })⊕h\left(E\right)$, thus each participant first checks the freshness of timestamps received and verifies whether the same timestamps are present in the transmitted messages. Hence, this design discards the possibility of replay attacks in our proposed scheme.

### 5.3. Resistance to Sensed Data Disclosure Attacks

In the access control and encryption phase of the proposed scheme, the sensed data *m* is embedded in the encrypted message M=m⊕SK, and *m* is well-protected via high-entropy session key $SK=h(E⊕a⊕T_{u})$. Here, we assume that $U_{a}$ can obtain the parameter *a* from a legal smart card and can eavesdrop the transmitted messages $<C,TID_{i},T^{\prime }>$ and <M> from the public channels between the user $U_{i}$ and the sensor nodes *S*. $U_{a}$ can use the collected parameters to compute $h(a||TID_{i}||T^{\prime })$ and $C⊕h(a||TID_{i}||T^{\prime })$ and derive h(E). However, without having the knowledge of secrets *E* and $T_{u}$, an attacker $U_{a}$ cannot derive SK from h(E) because of the irreversibility of the secure one-way hashing function.

On the other hand, during the login phase of the proposed scheme, we assume that the parameter $M_{i}=\alpha \times P_{0}$ and the public key $P_{pub}=S_{0}\times P_{0}$ of TA are disclosed. However, the secret parameter $N_{i}=\alpha \times P_{pub}=\alpha S_{0}P_{0}$ cannot be calculated by $U_{a}$ since the random number α is unknown due to the infeasibility of deriving them from $M_{i}$ by solving ECDLP. Moreover, during the access control and encryption phase, a unique and fresh secret parameter $N_{i}$ is computed in each new session using the random parameter α and the private key $S_{0}$. Due to the difficulties of ECDHP, $U_{a}$ cannot derive $N_{i}$ from $M_{i}$ and $P_{pub}$, and thus, the protection of fresh secret parameter $h(N_{i})$ does not allow $U_{a}$ to gain *E* and $T_{u}$ from RM. Therefore, $U_{a}$ cannot successfully derive *m* from *M* by computing $m=M⊕h(E⊕a⊕T_{u})$, and the confidentiality of the sensed data *m* is guaranteed in the proposed scheme.

### 5.4. Resistance to Sensed Data Forgery Attacks

In the access control and encryption phase of the proposed scheme, the sensor node *S* first authenticates the user $U_{i}$ by verifying whether $C^{\prime }=h(a||TID_{i}\left|\right|T^{\prime })⊕h\left(h\left(b⊕TID_{i}\right)\right)=C$. Due to the protection of using timestamp $T^{\prime }$ and the secret parameters *a* and $h(b⊕TID_{i})$, no one can forge a valid message $<C,TID_{i},T^{\prime }>$ to pass *S*’s verification. In addition, we assume that the attacker $U_{a}$ intercepts the response message *M* and tries to generate a legitimate message $M^{\prime }=m^{\prime }⊕h\left(E⊕a⊕T_{u}\right)$ with fake sensed data $m^{\prime }$. However, since $U_{a}$ does not know the secret parameters *E* and $T_{u}$, it cannot generate the legitimate message $<M^{\prime }>$. Thus, the proposed scheme could withstand the sensed data forgery attacks.

### 5.5. Resistance to Stolen Smart Card Attacks

Suppose that the smart card of $U_{i}$ is lost or stolen. The attacker $U_{a}$ could get the stored parameters $<Y_{i},X_{i},W_{i},a,E,q,P_{0},P_{pub},h\left(\cdot \right)>$ and try to impersonate $U_{i}$ to successfully login to the trusted authority TA. $U_{a}$ can first guess a candidate identity $ID_{i}^{*}$ and password $PW_{i}^{*}$ and compute $r^{*}=X_{i}⊕h(ID_{i}^{*}||PW_{i}^{*})$$V_{i}^{*}=W_{i}⊕h(ID_{i}^{*}||PW_{i}^{*}\left|\right|r^{*})$ and $Y_{i}^{*}=h(V_{i}^{*}\left|\right|r^{*}||h(ID_{i}^{*}||PW_{i}^{*}))$. The way for $U_{a}$ to learn $PW_{i}$ is to find out the correct pair $\left(ID_{i}^{*},PW_{i}^{*}\right)$ such that $Y_{i}=Y_{i}^{*}$. In the proposed scheme, we assume the probability of guessing $ID_{i}$ composed of exact *l* characters and $PW_{i}$ composed of exact *m* characters is approximately $\frac{1}{2^{6l+6m}}$. This probability is negligible, and $U_{a}$ has no feasible way to derive $ID_{i}$ and $PW_{i}$ of the user $U_{i}$ in polynomial time.

### 5.6. Resistance to Off-Line Password Guessing Attacks

In the proposed scheme, we assume that an attacker $U_{a}$ could eavesdrop all of the transmission messages $<M_{i},Q_{i},T_{L}>$, <RM>, $<C,TID_{i},T^{\prime }>$ and <M> between $U_{i}$, TA and *S*. However, neither the smart card, nor the transmission messages include $U_{i}$’s password $PW_{i}$. Therefore, the proposed scheme could withstand the off-line password guessing attack.

### 5.7. Provision of the Efficient Login Phase

In order to illustrate the verification mechanism during the login phase, three cases are taken into consideration. Case 1 assumed $U_{i}$ inputs a correct identity $ID_{i}$ and incorrect password $PW_{i}^{*}$. Case 2 assumed $U_{i}$ inputs an incorrect identity $ID_{i}^{*}$ and correct password $PW_{i}$. Case 3 assumed $U_{i}$ inputs incorrect identity $ID_{i}^{*}$ and incorrect password $PW_{i}^{*}$.

Case 1: After the user inputs $\left(ID_{i},PW_{i}^{*}\right)$, the smart card computes $r^{*}=X_{i}⊕h(ID_{i}||PW_{i}^{*})$, $V_{i}^{*}=W_{i}⊕h(ID_{i}||PW_{i}^{*}\left|\right|r^{*})$ and $Y_{i}^{*}=h(V_{i}^{*}\left|\right|r^{*}||h(ID_{i}||PW_{i}^{*}))$ and verifies $Y_{i}{\;}_{=}^{?}\;h(V_{i}^{*}\left|\right|r^{*}||h(ID_{i}||PW_{i}^{*}))$. In fact, the verification cannot pass as $Y_{i}\neq h(V_{i}^{*}\left|\right|r^{*}||h(ID_{i}||PW_{i}^{*}))$, and the smart card immediately terminates the session.
Case 2: After the user inputs $\left(ID_{i}^{*},PW_{i}\right)$, the smart card computes $r^{*}=X_{i}⊕h(ID_{i}^{*}||PW_{i})$, $V_{i}^{*}=W_{i}⊕h(ID_{i}^{*}||PW_{i}\left|\right|r^{*})$ and $Y_{i}^{*}=h(V_{i}^{*}\left|\right|r^{*}||h(ID_{i}^{*}||PW_{i}))$ and verifies $Y{\;}_{=}^{?}\;h(V_{i}^{*}\left|\right|r^{*}||h(ID_{i}^{*}||PW_{i}))$. Furthermore, the verification cannot pass as $Y\neq h(V_{i}^{*}\left|\right|r^{*}||h(ID_{i}^{*}||PW_{i}))$, and the smart card immediately terminates the session.
Case 3: After the user inputs $\left(ID_{i}^{*},PW_{i}^{*}\right)$, the smart card computes $r^{*}=X_{i}⊕h(ID_{i}^{*}||PW_{i}^{*})$, $V_{i}^{*}=W_{i}⊕h(ID_{i}^{*}||PW_{i}^{*}\left|\right|r^{*})$ and $Y_{i}^{*}=h(V_{i}^{*}\left|\right|r^{*}||h(ID_{i}^{*}||PW_{i}^{*}))$ and verifies $Y{\;}_{=}^{?}\;h(V_{i}^{*}\left|\right|r^{*}||h(ID_{i}^{*}||PW_{i}^{*}))$. Similarly, the verification cannot pass as $Y\neq h(V_{i}^{*}\left|\right|r^{*}||h(ID_{i}^{*}||PW_{i}^{*}))$, and the smart card immediately terminates the session.

### 5.8. Provision of User Anonymity

Based on the design of our proposed scheme, the excellent property of user anonymity can be guaranteed at every phase. We cleverly mask the real identity of $U_{i}$ via a public channel, and no attacker can compromise $U_{i}$’s real identity by launching security attacks. First, in the login phase, $U_{i}$’s real identity is included in $Q_{i}=h\left(N_{i}\right)⊕(ID_{i}\left|\right|O_{i})$. Thus, $U_{a}$ cannot reveal $Q_{i}$ without $h(N_{i})$. Additionally, in the verification and access control and encryption phases, the temporary identity $TID_{i}$ is generated and utilized to replace $U_{i}$’s identity transmitted among the user and the sensor nodes. That is to say, all of the identities are transmitted in cipher format instead of plaintext, and these temporary identities will be randomized at each new session. As a result, our proposed scheme can provide the property of user anonymity.

### 5.9. Provision of Mutual Authentication

In the login phase of the proposed scheme, only the legitimate user can know the secret parameter $V_{i}=h(ID_{i}\left|\right|S_{0}\left||a\right)$ to generate a legal $O_{i}$. Therefore, in Step 3 of the verification phase, TA can authenticate $U_{i}$ by checking if the decrypted $O_{i}$ is equal to the computed $O_{i}^{\prime }$. Moreover, in Step 5 of the verification phase, only the legal TA can own the secret key $S_{0}$ to compute the common secret parameter $h(N_{i})$. As a result, $U_{i}$ can authenticate TA by decrypting RM and checking if the revealed $ID_{i}$ is involved in RM.

On the other hand, in the access control and encryption phase, only the legal user can obtain the secret parameter h(E) to generate a legal *C*. Thus, in Step 4 of the access control and encryption phase, *S* can authenticate $U_{i}$ by checking if the received *C* is equal to the computed $C^{\prime }$. Additionally, in Step 5 of the access control and encryption phase, only the participated *S* can calculate the common session key $SK=h(E⊕a⊕T_{u})$ to encrypt the sensed data by computing M=m⊕SK. Finally, $U_{i}$ can also authenticate *S* by establishing the common session key SK and checking if the sensed data *m* are involved in *M* by decrypting m=M⊕SK.

### 5.10. Provision of Session Key Security

Since the common session key SK is only shared and established among the user $U_{i}$ and the sensor nodes *S*, in order to establish a secure and authenticated channel for late successive transmission, the session key SK not only ensures confidentiality, but also achieves authenticity of participants and messages. Based on the design of session key $SK=h(E⊕a⊕T_{u})$, *E* is used for verifying the integrity of the transmitted messages, whereas $T_{u}$ is used for preventing possible replay and misuse service attacks. As a result, the session key security and data confidentiality can be provided in the proposed authentication scheme.

## 6. Security Proof of the Proposed Scheme

Here, we follow similar techniques to demonstrate the security of our scheme in the random oracle model [27,28,29,30] and under the elliptic curve Diffie–Hellman problem (ECDHP).

### 6.1. Adversarial Model

We assume an adversary A is a probabilistic polynomial time algorithm and allowed to issue the following queries to some oracles. Note that an oracle has multiple instances $\prod_{U}^{j}$, where U denotes participants and j∈N. Here, we set $U\in \{U_{i},TA,S\}$ and may use A to simulate the proposed scheme via issuing queries.

1. $Send(\prod_{U}^{j},m)$ query: Upon receiving this query with message *m*, instance $\prod_{U}^{j}$ follows the proposed scheme and then returns the result to A.
2. $Hash(\prod_{U}^{j},m)$ query: Upon receiving this query with message *m*, instance $\prod_{U}^{j}$ returns a random value to A.
3. $Corrupt(\prod_{U_{i}}^{j},U)$ query: A may query user *U*’s password. Upon receiving this query, instance $\prod_{U_{i}}^{j}$ returns a password $PW_{U}$ to A. Note that this query models the forward secrecy of session key.
4. $Reveal(\prod_{E\in \{U_{i},S\}}^{j})$ query: A may query the previous established session keys. Upon receiving this query, instance $\prod_{E\in \{U_{i},S\}}^{j}$ returns a previous session key to A, if it has accepted. Otherwise, $\prod_{E\in \{U_{i},S\}}^{j}$ returns a random string to A. Note that this query models the knowing the session key attack of session key.
5. $Test(\prod_{E\in \{U_{i},S\}}^{j})$ query: A may only issue this query once. Upon receiving this query, instance $\prod_{E\in \{U_{i},S\}}^{j}$ flips an unbiased coin *b*. If b=1, it returns a session key. Otherwise, it returns a random string. Note that this query models the semantic security of session key.

### 6.2. Mutual Authentication between $U_{i}$ and TA

**Theorem** **1.** In the random oracle model, assume that there exists an adversary A with a non-negligible advantage $\epsilon_{0}$ that can impersonate $U_{i}$ to communicate with TA. Then, there is a challenger C, which can solve the elliptic curve Diffie–Hellman problem (ECDHP) with advantage $q\cdot \epsilon_{0}<\epsilon \leq \frac{q_{H}}{2^{k}}$, where $q_{S}$ denotes the maximum number of send queries issued by A, $q_{H}$ denotes the maximum number of hash queries issued by A and k denotes the length of the hash value.

**Proof.**  Note that we say that A successfully impersonates $U_{i}$ to communicate with TA. This means that TA accepts $\left(M_{i},Q_{i},T_{L}\right)$, but it has not been produced by $U_{i}$. In this case, it could be that A guessed $\left(M_{i},Q_{i},T_{L}\right)$. Then, this leads to:

(1) $$\epsilon_{0}<\frac{q_{S}}{q}\times \mathrm{Pr}[O_{i}=h(ID_{i}\left|\right|V_{i}||TL)|ID_{i}\left|\right|O_{i}=Q_{i}⊕h\left(N_{i}^{\prime }\right);V_{i}=h(ID_{i}\left|\right|S_{0}\left||a)\right]\times \frac{1}{q_{S}}\leq \frac{q_{S}}{q}\times \frac{q_{H}}{2^{k}}\times \frac{1}{q_{S}}.$$

Given that $M_{i}=a\cdot P$ and $P_{pub}=b\cdot P$ to A for *a*, $b\in Z_{q}^{*}$ are unknown, then, A can compute $N_{i}^{\prime }=abP$. Thus, given $\left(P,M_{i},P_{pub}\right)=\left(P,aP,bP\right)$, C can use A as a subroutine to compute abP. In other words, C can solve ECDLP with the advantage $q\cdot \epsilon_{0}<\epsilon \leq \frac{q_{H}}{2^{k}}$. ☐

**Theorem** **2.** In the random oracle model, assume that there exists an adversary A with a non-negligible advantage $\epsilon_{1}$ that can impersonate TA to communicate with $U_{i}$. Then, there is a challenger C, which can solve the elliptic curve Diffie–Hellman problem (ECDHP) with advantage $\epsilon \geq \epsilon_{0}-\frac{1}{2^{k}}-\frac{q_{S}^{2}\cdot q_{H}^{2}}{q\cdot 2^{k}}$, where $q_{S}$ denotes the maximum number of send query issued by A, $q_{H}$ denotes the maximum number of hash query issued by A and k denotes the length of the hash value.

**Proof.**  Without of loss generality, we assume that the event that violates $U_{i}$-to-TA authentication denoted by $Event^{U_{i}2TA}$ does not occur. Similarly, we use the symbol $Event^{TA2U_{i}}$ to define the event that violates TA-to-$U_{i}$ authentication. We say that A successfully impersonates TA to communicate with $U_{i}$. This means that at some point, $U_{i}$ accepts RM after sending $\left(M_{i},Q_{i}\right)$. However, RM has not been produced by TA. In this case, it could be the following three cases:

1. A guessed RM. The probability of this case is $\frac{1}{2^{k}}$.
2. $M_{i}$ and $Q_{i}$ were obtained in other session. The probability of this case is $\frac{q_{S}\cdot \left(q_{S}-1\right)}{q}\times \frac{q_{H}\cdot \left(q_{H}-1\right)}{2^{k}}$ less than $\frac{q_{S}^{2}\cdot q_{H}^{2}}{q\cdot 2^{k}}$.
3. A had issued the hash query for $N_{i}^{\prime }$.

Thus, we have:

(2) $$\mathrm{Pr}\left[Event^{TA2U_{i}}|\neg Event^{U_{i}2TA}\right]\leq \mathrm{Pr}[RM=h\left(N_{i}^{\prime }\right)⊕(ID_{i}||TID_{i}\left|\right|T_{u}\left||E)\right]+\frac{1}{2^{k}}+\frac{q_{S}^{2}\cdot q_{H}^{2}}{q\cdot 2^{k}}.$$

Given $M_{i}=a\cdot P$ and $P_{pub}=b\cdot P$ to A for *a*, $b\in Z_{q}^{*}$ are unknown, then, A can compute $N_{i}^{\prime }=abP$. Thus, given $\left(P,M_{i},P_{pub}\right)=\left(P,aP,bP\right)$, C can use A as a subroutine to compute abP. In other words, C can solve ECDLP with the advantage $\epsilon \geq \epsilon_{0}-\frac{1}{2^{k}}-\frac{q_{S}^{2}\cdot q_{H}^{2}}{q\cdot 2^{k}}$.

### 6.3. *S* Authenticates $U_{i}$ and Key Agreement

**Theorem** **3.** Under the elliptic curve computational Diffie–Hellman problem (ECDHP), no adversary can impersonate user $U_{i}$ to communicate with sensor node S after $U_{i}$ is authenticated as a legal user by TA.

**Proof.**  No one can forge $C=h(a||TID_{i}||T^{\prime })⊕E$ except legal user $U_{i}$ because *a* is a secret value stored in $U_{i}$’s smart card, and *E* is obtained from the procedures of $U_{i}$ authenticating TA. By Theorem 2, we have proved that no one can impersonate TA to communicate with $U_{i}$ under the ECDHP. Even if the $U_{i}$’s smart card is broken, the adversary is still unable to forge *E*. ☐

**Theorem** **4.** Under the elliptic curve computational Diffie–Hellman problem (ECDHP), only user $U_{i}$ and sensor node S can establish a session key SK after $U_{i}$ is authenticated as a legal user by TA. In other words, no adversary can compute SK except $U_{i}$ and S.

**Proof.**  According to the proofs of Theorems 2 and 3, no one can compute $SK=h(E⊕a⊕T_{u})$ except $U_{i}$, an authenticated legal user. In another aspect, only *S* can compute SK because TA sends *a* and $\left(T_{u},b,TID_{i}\right)$ to *S* via a secure channel, and *E* is computed by $h(b⊕TID_{i})$. ☐

## 7. Performance Analyses and Comparisons

In this section, we provide a performance comparisons among our scheme and two existing ECC-based authentication schemes [5,21] for wireless healthcare sensor networks in terms of computation costs in the authentication process (which includes the login, verification, and access control and encryption phases). According to the experimental results of He [31], the execution times are given in Table 1, where the hardware platform is a Pentium IV 3-GHz processor with library MIRACL [32]. As shown in Table 1, it is clear that the elliptic curve scalar point multiplication and the bilinear pairing operation are more complicated than other operations, and the running time of the addition operation of points, the map-to-point hash function and the one-way hash function could be ignored. Therefore, we only need to count the execution time of the elliptic curve scalar point multiplication and the bilinear pairing operation.

In Table 2, we summarize the efficiency comparisons among our proposed scheme and other previous WSN-based authentication schemes in terms of computational complexity and the execution time, where the total execution times are measured using Table 1. From Table 2, we can see that the computation cost of our scheme is lower than that of Yeh et al.’s and Liu–Chung’s schemes on both the user, the trusted authority and the sensor node side. Therefore, our proposed scheme is the most efficient compared to the other two related schemes in terms of overall computation costs, and it can be claimed that the execution time of the proposed scheme is suitable for different real-life applications, including medical care systems.

Lastly, the security criteria and functional properties of three ECC-based authentication schemes are summarized in Table 3. It is visible from Table 3 that Yeh et al.’s scheme [5] is vulnerable to password disclosure attack in the registration phase and also does not provide the user anonymity property, where Liu–Chung’s scheme [21] does not support this property. The proposed scheme can prevent all of the security weaknesses of the former scheme and provide mutual authentication and user anonymity to protect data integrity and user privacy. From Table 2 and Table 3, the proposed scheme not only keeps lower computational cost, but also possesses more security requirements along with strong security protection on the relevant security attacks for IoT-based medical care systems.

## 8. Conclusions

In this paper, we first give a brief review of Liu–Chung’s authentication scheme combined with its basic security analysis and find that their scheme is vulnerable to password disclosure, off-line password guessing, sensed data disclosure, sensed data forgery, replay attacks and the stolen smart card problem. Furthermore, their scheme cannot achieve user anonymity and session key security, and it has unnecessary redundancy in protocol design. In order to repair their security flaws and improve the system performance, an improved efficient scheme is proposed. The security analysis indicates that the proposed authentication scheme is able to withstand those attacks mentioned and satisfies all desirable security attributes, such as user anonymity, mutual authentication, session key security and an efficient verification mechanism during the login phase. Comparing the efficiency with other ECC-based authentication schemes, the proposed scheme is comparable in terms of the computational overheads and practical as the secure authentication mechanism for the IoT-based medical care system.

## Figures and Tables

**Table 1:** Execution time (in milliseconds; ms) of various cryptographic operations.

Notations	Descriptions
$T_{EM}$	The time of executing an elliptic curve scalar point multiplication, 1$T_{EM}\approx$ 1.17 ms
$T_{BP}$	The time of executing a bilinear pairing operation, 1$T_{BP}\approx$ 3.16 ms
$T_{EA}$	The time of executing an addition operation of points, 1$T_{EA}<0.1$ ms, which is negligible
$T_{MH}$	The time of executing a map-to-point hash function, 1$T_{MH}<1$ ms, which is negligible
$T_{H}$	The time of executing a one-way hash function, 1$T_{H}<0.01$ ms, which is negligible

**Table 2:** Performance comparisons among the proposed scheme and other related schemes.

	Yeh et al. [5] (2011)	Liu–Chung [21] (2016)	The Proposed Scheme
Computation cost ($U_{i}$)	2$T_{EM}$ + 1$T_{EA}$ + 1$T_{MH}$ + 3$T_{H}$	1$T_{EM}$ + 1$T_{BP}$ + 1$T_{MH}$ + 2$T_{H}$	2$T_{EM}$ + 8$T_{H}$
Computation cost (TA)	5$T_{EM}$ + 3$T_{EA}$ + 4$T_{MH}$	2$T_{BP}$ + 1$T_{H}$	1$T_{EM}$ + 4$T_{H}$
Computation cost (*S*)	2$T_{EM}$ + 2$T_{EA}$ + 3$T_{MH}$	1$T_{BP}$ + 1$T_{MH}$ + 2$T_{H}$	4$T_{H}$
Total execution time	10.53 ms	13.81 ms	3.51 ms

**Table 3:** Functionality comparisons among the proposed scheme and other related schemes.

	Yeh et al. [5] (2011)	Liu–Chung [21] (2016)	The Proposed Scheme
F1	χ	χ	√
F2	√	χ	√
F3	√	√	√
F4	√	χ	√
F5	χ	χ	√
F6	√	χ	√
F7	−	χ	√
F8	−	χ	√
F9	√	χ	√
F10	√	χ	√

F1: Provision of user anonymity; F2: provision of efficient login phase; F3: provision of mutual authentication; F4: provision of session key security; F5: prevention of password disclosure attack; F6: prevention of replay attack; F7: prevention of sensed data disclosure attack; F8: prevention of sensed data forgery attack; F9: prevention of stolen smart card attack; F10: prevention of off-line password guessing attack; √: yes; χ: no; −: not mentioned.
